# Exploring the Experience of Breastfeeding Among Working Mothers at Healthcare Facility in Saudi Arabia: A Qualitative Approach

**DOI:** 10.7759/cureus.25510

**Published:** 2022-05-31

**Authors:** Hala AlSedra, Alaa A AlQurashi

**Affiliations:** 1 Patient Education Department, King Fahad Medical City, Riyadh, SAU; 2 Epidemiology and Public Health Department, King Fahad Medical City, Riyadh, SAU

**Keywords:** maternity leave, saudi arabia, working mothers, interpretative phenomenological analysis, exclusive breastfeeding

## Abstract

Background: Breastfeeding has both short- and long-term benefits for infants, mothers, the environment, healthcare costs, and wider society. In Saudi Arabia, breastfeeding has undergone a considerable decline in recent decades due to population changes and developing socioeconomic status. Limited research studies have explored the relationship between work and breastfeeding in Saudi Arabia. Nevertheless, most research has mentioned employment as the factor having the largest effect on breastfeeding in Saudi Arabia.

Purpose: The aim of this study is to explore the factors that influence breastfeeding among working mothers in a tertiary hospital.

Methods: Using a phenomenological qualitative design, nine working women were recruited through purposive sampling at King Fahad Medical City (KFMC), Saudi Arabia in 2019. A semi-structured interview of 30-50 min was conducted with each participant. Thematic analysis was used to analyze the data.

Results: Most women talked about the difficulties of combining breastfeeding with work, which resulted in the early discontinuance of breastfeeding before six months. The findings show that the key factors that affect breastfeeding include maternal attributes such as knowledge about breastfeeding, prenatal decisions, and mothers’ conflicting priorities, as well as the availability of social and workplace support. These influenced whether working women in healthcare settings in Saudi Arabia were able to continue breastfeeding while employed.

Conclusion: This study highlights the importance of issues and problems faced by working mothers throughout postpartum. Inventive solutions need to be developed to facilitate breastfeeding in the work environment. This will give working women the option to breastfeed for a longer period to improve the health of both themselves and their babies. The availability of a private place and support from employers to utilise the institutional policy will enable working women to continue breastfeeding after returning to work.

## Introduction

Breastfeeding is the optimal and most natural way of nurturing infants. Many professional health organizations, including the United Nations Children’s Fund (UNICEF) and the World Health Organization [1], have strongly endorsed practicing breastfeeding exclusively for the first six months of life [2].

Breastfeeding has numerous benefits, including improving child health, nutrition, development, and survival, especially in developing countries. It also creates a strong bond between the mother and the child, which can have long-term positive repercussions regarding motivation, behavior, feelings of wellbeing, and security. Breastfeeding, therefore, has short- and long-term benefits for newborn children, mothers, the environment, health service costs, and wider society [3,4].

However, in Saudi Arabia, exclusive breastfeeding rates have declined over the years from 90% to 50% at the age of three months [5]. Furthermore, the frequency with which breastfeeding has continued for as long as two years has fallen from 32% to 3.2% over the last two decades [6].

Maintaining the rhetoric and social policy choices may have a further effect on current breastfeeding rates. Many studies in Saudi Arabia have shown the importance of breastfeeding education for Saudi women in improving rates of breastfeeding in society [7,8]. Barriers to breastfeeding reported by mothers included lack of knowledge, family economic status, social support, embarrassment, employment, childcare, and barriers related to health services [9].

A recent local phenomenological study clarified factors associated with breastfeeding practice among Saudi mothers, such as professional training and education, friendly hospital initiative accreditation, and improving conditions at workplaces [10].

Nevertheless, planning for breastfeeding promotion requires an acknowledgment of the present circumstances surrounding infant nourishing and related factors through well-planned observational investigations. A mother's work status is usually reported as the factor most frequently affecting whether mothers exclusively breastfeed [11,12]. However, there is insufficient data to monitor progress and develop promotion programs at the workplace. Additionally, there has been inadequate discussion of the important factors that affect a mother who works outside the home.

This study aimed to explore the experience of exclusive breastfeeding during work and to define any barriers that hinder their intentions to breastfeed on returning to work and any support they have received to help them overcome these difficulties.

## Materials and methods

A qualitative approach allows researchers to explore in-depth aspects of phenomena based on individual experiences to bridge gaps in research or expand on existing knowledge. The research focused on working mothers in Saudi Arabia and was conducted at a tertiary hospital in Riyadh, Saudi Arabia. King Fahad Medical City (KFMC) in 2019. This hospital is the largest medical facility and is typical of Saudi healthcare institutions.

Sample profile

The sample comprised nine young mothers aged between 27 and 34, all of whom had a level of educational attainment ranging from bachelor’s degrees to postgraduate degrees. All are full-time workers. The sample includes different types of working mothers. Five women were located in clinics; four women performed administrative work, and one of the women had shift responsibilities. To better explore the experience of breastfeeding while working, the sample includes the common working hours. Eight women worked eight to nine hours per day, and one woman reported working five to nine.

The inclusion and exclusion criteria for participants in this study are presented in Table 1.

**Table 1:** The inclusion and exclusion criteria of the mothers.

Inclusion criteria	Exclusion criteria
Postpartum working mothers, who work at KFMC.	Mothers who have never experienced breastfeeding.
Mothers with a live infant aged between 6 and 24 months.	Mothers whose last delivery was more than two years ago.
Mothers who had been practicing breastfeeding for at least one month.	Mothers who left work to become a housewife after giving birth.

Three of the women were first-time mothers, and this was their first chance to breastfeed. Four were second-time mothers, and two were mothers for the third time. Mothers’ and infants’ characteristics are presented in Table 2.

**Table 2:** Mothers’ and infants’ characteristics.

Mothers’ characteristics				Infants’ characteristics		Breastfeeding characteristics	
	Age (years)	Number of children	Years of experience at KFMC	Age (months)	Gender	Duration of exclusive BF	Duration of BF
1	31	2	10 y	1 y and 10 m	Male	1 month and 1 week	2 months
2	29	2	7 m	11 m	Male	0	2 months
3	28	1	4 y	1 y and 5 m	Female	First day	3 months
4	27	1	2 y	7 m	Male	3 months	Until now
5	29	1	6 y	1 y and 6 m	Male	0	2 months
6	30	2	6 y	7 m	Female	4 months	5 months
7	34	3	12 y	4 m	Male	0	0
8	28	1	4 y	1 y and 3 m	Female	3 days	4 months
9	33	3	11 y	1 y and 3 m	Male	2 months and 1 week	8 months

Sample recruitment and interview

The participants were recruited through a non-probability sampling method. Recruitment took place by sending emails in the postmaster's internal system to 100 employees to request volunteers eligible for the research by the snowball sampling technique. Nine interested mothers replied, then they were provided with more details on the research objective and procedure. According to Giorgi [13], there is no specific requirement for sample size in qualitative research; however, a minimum of at least three participants is recommended.

Then, a follow-up email was sent requesting a phone number so that a proper date, time, and location could be chosen in which to conduct the interview. Confirmation via a phone message was sent prior to the interview. The recruitment period continued for two months from July to August 2019. Semi-structured interviews were conducted with nine mothers in face-to-face environments, such as a participant’s office or a private meeting room. Each interview lasted between 30 and 50 minutes.

Data analysis

The data analysis was conducted in accordance with guidance on Interpretative Phenomenological Analysis (IPA) using NVivo software (QSR International, Melbourne, Australia) [14]. The analysis process has four steps, including transcription of the audio recordings, reviewing, coding agreement, and theme identification. All modifications were applied to the transcribed interviews before the analysis began. The interviews were audio-recorded and transcribed literally in the Arabic language, following which they were translated into English by experts in the English language. The interview transcripts were then integrity-checked by experts in the areas of English language and health sciences. Following integrity checking, the transcripts were anonymised by replacing names, places, and identifying information with pseudonyms. The researcher coded the data line by line and then developed and grouped emerging themes. The results were then discussed with the research, as a result of which themes that did not meet the aims of the research were rejected. The coding also helped to define both semantic and latent content. Semantic themes can be found on the surface, while latent themes can be deduced at an interpretative level [15]. The researchers worked together after the themes were identified to re-enhance the coding frame through the renaming, removal, and separating out of codes.

Ethical approval

An application for ethical approval was also submitted to the KFMC ethical committee and was approved with log number 19-229E. Participants were informed they could withdraw from the study at any time. The researcher also reassured the participants regarding the confidentiality of the data and respect for their privacy. All participants and their children, husbands, service providers such as doctors and other family members were assigned pseudonyms. Additionally, all discussion of locations, departments, or other places that could reveal a participant’s identity was replaced with pseudonyms or other fictitious representations to ensure the privacy of the participants.

## Results

The place of breastfeeding in the medical city includes rooms at the women's hospitals next to admitted patients and rooms at hospitals’ daycare. The breastfeeding policy at the institution is to have one hour only for breastfeeding, and it has to be either the first hour in the morning or the last hour of your work shift. Mothers received conflicting information about how the work environment influences breastfeeding. Some reported positive reactions from the work environment, whereas others received negative reactions towards their efforts at breastfeeding. In the workplace environment, the mothers highlighted the significance of support from supervisors, facilities, co-workers, friendly mothers, and job flexibility as important facilitators of continued breastfeeding at work.

Theme 1: physical facilities

Assessing Specified Breastfeeding Spaces

The significance of having a private place to breastfeed was mentioned by all interviewed mothers. They highly expressed the need to close doors to feel safe when expressing and breastfeeding. One woman referred to the element of feeling safe as the ability to avoid being seen by her colleagues and patients while expressing breast milk.

"...I think these rooms are open for patients and staff together. In my opinion, the staff should have separate rooms because I will not feel comfortable breastfeeding if I find a patient there..." (Riham)

Most of the women who could enter the private places were not satisfied with the facilities provided, characterizing them as uncomfortable, small, and lacking features such as a fridge. This experience was described by more than one mother as follows:"... the room has only two sofas and one curtain covers these two sofas, and all the mothers sitting together..." (Arwa)

Accessing Available Facilities

Some women were unaware that there were rooms on KFMC that were used as spaces specifically for expressing breast milk. For instance, one mother said:"... Unfortunately, I did not know about the expression rooms available in the workplace. I have two kids and I just heard about it from you. These rooms have not been announced at work..." (Jamila).

Among working mothers who were aware of the facilities available, a number reported challenges locating and accessing the spaces. "...There is one place available to express breast milk, which is in the women’s hospital, but, unfortunately, I can't go every two hours to express milk there. It is too difficult to be in the main hospital where I usually work and move every two hours to the women’s hospital to express milk..." (Dima).

A lack of awareness of the available facilities, an inability to access them, or a disappointment with the equipment available, led some mothers to express milk in other parts of the workplace, such as staff rooms, clinics, prayer rooms, and female waiting areas. One participant said, "…I already have the pumping machine. Then why should I go to the expression room in the women’s hospital? So, after I finish my clinic, I lock the door and start to express... " (Sarah).

In the same vein, "...We did not have any milk expression rooms (in her department), therefore I started to express my breast milk in the toilet..." said (Nada). When asked about the reason for choosing toilets, she said:"...I found privacy in the toilets and it was close to my workplace…" (Nada).

Having a private office creates a positive work environment for breastfeeding mothers to express milk. Those working mothers who had a private office were often aware of the advantages of this and remarked: "...At work, I usually sit in my office at the break time and express breast milk, then I put the milk in the fridge located in my office..." (Bunia).

Having a place for breastfeeding at daycare is basic support that should be provided to the mothers, especially if they are paying fees to the daycare. Easy access and availability of rooms are considered a main factors in continuing breastfeeding.

"…Even if the workplace provides expression rooms, the expression may reduce the mother’s milk flow. Mothers need to have direct bonding with their children, not only through pumping milk. I feel this issue can be solved through daycare…" (Jamila).

"…The daycare has a very long waiting list extending to a year and two years, there is no chance to breastfeed (at work). There is no facilitation of this in the workplace…" (Arwa).

Theme 2: job flexibility

Participants explained how the absence of job flexibility and the non-availability of time to express breast milk at work are different concerns confronted by mothers in the workplace environment. When sharing thoughts about job flexibility in the workplace with those who do not have a family member to take care of their child, one mother stated: "…The administration does not take into consideration that you are a new mother and you have responsibilities. The work duties and pressure remain the same as in the past, as do the working hours. There was no support; therefore, I quit breastfeeding within two weeks after returning to work…" (Nada).

Theme 3: work policies on breastfeeding

Maternity leaves are considered acceptable because mothers can take up to 70 days off. However, participants argued that maternity leave at KFMC did not empower them to continue breastfeeding for longer duration; they felt that maternity leave of only 70 days was insufficient. "...The breastfeeding hour is not logical. How many times will you feed your child within nine hours of work? At least four times if we count it, every two hours a feed..." (Jamila).

When asked about the reason for stopping breastfeeding after returning to work, one mother said: "...long working hours make you non-portable to breastfeed, and the shortness of maternity leave makes you return to work, and you are not ready or well prepared for anything…" (Bunia).

Another mother said: "...With my first baby, my leave was shorter, but with the second baby I had a longer leave, which allowed me to breastfeed for a longer time." When asked about things that affected her decision regarding breastfeeding when returning to work, she said: "…The first thing is the working hours. Second is the one-hour breastfeeding, which is considered a short period and I should take it at the beginning or by the end of work. The last thing is that there is no available place to bring my child with me…" (Jamila).

Theme 4: supervisor support

Having the support of supervisors, managers, and colleagues was a positive factor that support mothers’ breastfeeding experiences, "…I asked for the breastfeeding hour and my supervisor was so very nice. He said it is not in the regulations for employees and also that I am not obliged to give it to you, but I will. You can take it on days where you have a full nine-hour working day..." (Zina).

Discouragement and criticism from administrators and colleagues, on the other hand, were seen as the strongest factor that may influence the continuation of breastfeeding with employment. One of the mothers, whose supervisor was not supportive of her breastfeeding, said: "…I had problems with my supervisor regarding breastfeeding hour. My supervisor started to show me the regulations and explained that she had the right to refuse my request for a breastfeeding hour. I can take it in the middle of the work time, but the nature of my work does not allow me to take two hours for breastfeeding and a break hour. In addition, my daughter was not with me in the hospital, and I do not have the time to return home to feed her and return to work in those two hours…" (Nada).

For women who did not have help, breastfeeding was an immense challenge. For instance, the same mother said: "…I had requested unpaid leave after my maternity leave but my supervisor rejected it. The human resources were surprised as this was the first case they received where the supervisor refused maternity unpaid leave…" (Nada).

The same mother said: "…My administration thinks that mothers are very sensitive and try to manipulate the system…" (Nada).

## Discussion

The findings from this study encompassed four areas: (i) determining mothers’ experiences with breastfeeding since returning to work; (ii) defining the barriers affecting their intentions regarding breastfeeding when returning to work, and whether they received any support from others to help overcome these difficulties; and (iii) identifying whether women were aware of the support and policies provided by KFMC for lactating employees and their thoughts in relation to these.

In the current study, the data showed that support and facilities to express milk or breastfeed are available for mothers in the workplace, and this was associated with increased breastfeeding compared with working mothers who did not know whether these facilities existed or were available. Consequently, knowledge of these policies will positively influence breastfeeding.

Two of the mothers supported the idea of flexible scheduling for lactating workers. Some mothers complained that breast milk had to be pumped multiple times during working hours and that the break was inadequate to both pumps and have lunch, while others said that it was "difficult to leave the clinic" or that there was an "unpredictable workload" that made pumping difficult during working hours. The data indicated that the main work-related problem was that long and busy working hours did not allow the mother to express milk at work or necessitated leaving early to directly breastfeed their infant. Almost half of the participants intending to breastfeed at work gave the same reasons. Researchers referenced policies supportive of breastfeeding in the workplace, such as a formal breastfeeding period, milk expression, privacy, expert consultation, flexibility of workplace schedule, and childcare facilities [9,16,17], and reported that Saudi working mothers received eight weeks' paid maternity leave. They also found that mothers with longer maternity leaves were breastfeeding more than mothers with shorter maternity leaves [18]. Furthermore, a longer duration of maternity leave is more consistent with a longer duration of breastfeeding [19].

This study showed that workplace policies are related to working mothers' initial commencement of formula feeding or the early cessation of breastfeeding. Working mothers who do not have any access to breastfeeding support policies are more likely to completely discontinue breastfeeding shortly after returning to work than mothers who benefit from such a policy. Most working mothers who were not supported in the workplace decided to stop breastfeeding and start using formula milk for their infants. Working mothers may have access to policies that offer support for expressing milk, such as breastfeeding hours; however, these policies have to be approved by the supervisor. A work environment with supportive supervisors is important for achievement and success [20,21].

Therefore, the factor that could explain mothers’ attitude to breastfeeding is a lack of workplace support. Mothers in this study who had managers or supervisors who were used to working with breastfeeding mothers or had been through the same experience received more support than mothers with managers without such experience. Supporting this association, a few studies have shown that the absence of work environment support acts as a barrier to breastfeeding continuation and thus results in the early discontinuance of breastfeeding. By contrast, the positive outcomes of support for workplace breastfeeding include improving mothers’ ability to continue breastfeeding alongside employment [22]. In general, however, it is difficult for working mothers in the Saudi work environment to continue breastfeeding practices due to a lack of support. In the same vein, a qualitative study conducted among Pakistani mothers found that, regardless of the authenticated maternity leave policy at the institution, mothers could not benefit from such leave unless their employers granted permission [23]. The same issue emerged in this research as one of the mothers requested a breastfeeding hour and this was rejected by her supervisor. Mothers who have more support and control over their workplace conditions are more likely to be successful in accomplishing their decision to breastfeed while employed [24,25]. Regardless of the policies of the workplace, what affected mothers’ practice and intention with respect to breastfeeding was their aim in satisfying their intended behaviour. However, there was a range of beliefs among working mothers about breastfeeding or breast milk as a choice for nourishing their newborn child. In some cases, the mother’s choice changes over time in response to their environmental influences.

The availability of workplace support and physical facilities was useful in making a return to work more manageable. However, the characteristics of women, the level of family support, and the challenges they faced also influenced their infant feeding practices.

This study was strengthened by using a qualitative design for the first time to explore this topic in Saudi Arabia. The data collected were detailed and extremely useful, but were also subjective in nature. Obtaining the sensitive data through a discussion between the researcher and participant proved to be relatively easy. As a result, the data regarding women’s opinions, experiences, and beliefs were both rich and highly informative. Although the sample size was comparatively small, there were many similarities in the women’s experiences. Healthcare providers could therefore utilise the findings to promote breastfeeding among working women in hospitals using techniques focused on the mothers, their social network, and the wider community. However, qualitative data has certain limitations as the data are not generalizable. This study only focused on women who worked in KFMC; women who work in other organisations were not included. The study also excluded women who stopped working after giving birth.

## Conclusions

In Saudi Arabia, barriers to breastfeeding among mothers in the work environment have not been clearly recognised and measured. In light of the current outcomes, further support is needed to enable continued breastfeeding. Healthcare professionals should increase the awareness and attention of people forming part of the social network of working mothers with regard to providing support for breastfeeding, including child-care staff and family members. A supportive workplace environment can foster the motivation and confidence of mothers to breastfeed. Strict legislation and financial support, for example, might be introduced in the workplace in the future. In addition, mixed methods research, including qualitative and quantitative approaches, is needed to better understand barriers to breastfeeding.

## References

[1] Victora CG, Bahl R, Barros AJ (2016). Breastfeeding in the 21st century: epidemiology, mechanisms, and lifelong effect. The lancet.

[2] World Health Organization. (2011 (2022). Infant and young child feeding. https://www.who.int/en/news-room/fact-sheets/detail/infant-and-young-child-feeding.

[3] Borkhoff CM, Dai DW, Jairam JA (2018). Breastfeeding to 12 mo and beyond: nutrition outcomes at 3 to 5 y of age. Am J Clin Nutr.

[4] Scime NV, Patten SB, Tough SC, Chaput KH (2022). Maternal chronic disease and breastfeeding outcomes: a Canadian population-based study. J Matern Fetal Neonatal Med.

[5] Al-Sekait M (1988). A study of the factors influencing breastfeeding patterns in Saudi Arabia. Saudi Med J.

[6] Saied H, Mohamed A, Suliman A, Al Anazi W (2013). Breastfeeding knowledge, attitude and Barriers among Saudi women in Riyadh. J Nat Sci Res.

[7] Alyousefi NA (2021). Determinants of successful exclusive breastfeeding for Saudi mothers: social acceptance is a unique predictor. Int J Environ Res Public Health.

[8] Hegazi MA, Allebdi M, Almohammadi M, Alnafie A, Al-Hazmi L, Alyoubi S (2019). Factors associated with exclusive breastfeeding in relation to knowledge, attitude and practice of breastfeeding mothers in Rabigh community, Western Saudi Arabia. World J Pediatr.

[9] Lauer EA, Armenti K, Henning M, Sirois L (2019). Identifying barriers and supports to breastfeeding in the workplace experienced by mothers in the new hampshire special supplemental nutrition program for women, infants, and children utilizing the total worker health framework. Int J Environ Res Public Health.

[10] Murad A, Renfrew MJ, Symon A, Whitford H (2021). Understanding factors affecting breastfeeding practices in one city in the Kingdom of Saudi Arabia: an interpretative phenomenological study. Int Breastfeed J.

[11] Alzaheb RA (2017). A review of the factors associated with the timely initiation of breastfeeding and exclusive breastfeeding in the Middle East. Clin Med Insights Pediatr.

[12] Amin T, Hablas H, Al Qader AA (2011). Determinants of initiation and exclusivity of breastfeeding in Al Hassa, Saudi Arabia. Breastfeed Med.

[13] Giorgi A (2021). The descriptive phenomenological method in psychology: a modified Husserlian approach. APA PsycNET.

[14] Smith FP, Flower P, Larkin MJ (225). Interpretative Phenomenological Analysis: Theory, Method and Research.

[15] Creswell JW, Poth CN (2016). Qualitative Inquiry and Research Design: Choosing Among Five Approaches. https://www.worldcat.org/title/qualitative-inquiry-research-design-choosing-among-five-approaches/oclc/1140378606?referer=di&ht=edition.

[16] Salganicoff A (2018). The importance of strengthening workplace and health policies to support breastfeeding. Breastfeed Med.

[17] Holla-Bhar R, Iellamo A, Gupta A, Smith JP, Dadhich JP (2015). Investing in breastfeeding - the world breastfeeding costing initiative. Int Breastfeed J.

[18] Van Niel MS, Bhatia R, Riano NS (2020). The impact of paid maternity leaves on the mental and physical health of mothers and children: a review of the literature and policy implications. Harv Rev Psychiatry.

[19] Mirkovic KR, Perrine CG, Scanlon KS (2016). Paid maternity leave and breastfeeding outcomes. Birth.

[20] Scott VC, Taylor YJ, Basquin C, Venkitsubramanian K (2019). mpact of key workplace breastfeeding support characteristics on job satisfaction, breastfeeding duration, and exclusive breastfeeding among health care employees. Breastfeed Med.

[21] Snyder K, Hansen K, Brown S, Portratz A, White K, Dinkel D (2018). Workplace breastfeeding support varies by employment type: the service workplace disadvantage. Breastfeed Med.

[22] Nakada K (2021). Effectiveness of a breastfeeding program for mothers returning to work in Japan: a quasi-experimental study. Int Breastfeed J.

[23] Hirani SA, Karmaliani R (2013). Evidence based workplace interventions to promote breastfeeding practices among Pakistani working mothers. Women Birth.

[24] Wallenborn JT, Perera RA, Wheeler DC, Lu J, Masho SW (2019). Workplace support and breastfeeding duration: The mediating effect of breastfeeding intention and self-efficacy. Birth.

[25] Vilar-Compte M, Hernández-Cordero S, Ancira-Moreno M, Burrola-Méndez S, Ferre-Eguiluz I, Omaña I, Pérez Navarro C (2021). Breastfeeding at the workplace: a systematic review of interventions to improve workplace environments to facilitate breastfeeding among working women. Int J Equity Health.

